# Mono‐TKI and TKI Plus ICI in Unresectable Hepatocellular Carcinoma Progression on First‐Line Treatment of Lenvatinib: A Real‐World Study

**DOI:** 10.1002/cam4.70711

**Published:** 2025-03-06

**Authors:** Xue Yin, Na Deng, Jinglong Chen, Xiaoyan Ding

**Affiliations:** ^1^ Department of Cancer Center, Beijing Ditan Hospital Capital Medical University Beijing China

**Keywords:** hepatocellular carcinoma, immune checkpoint inhibitor, Lenvatinib, second‐line therapy, tyrosine kinase inhibitor

## Abstract

**Background:**

Lenvatinib (LEN) is the recommended first‐line therapy for unresectable hepatocellular carcinoma (uHCC), but resistance frequently develops, and limited data exist on second‐line treatments. This study evaluated the efficacy and safety with a focus on the sorafenib (SOR) or regorafenib (REG)‐ based monotherapy or combination therapy in patients with uHCC after failure of first‐line LEN.

**Methods:**

Patients with first‐line LEN failure between May 2018 and December 2023 were retrospectively collected. Based on second‐line regimens, 70 patients were divided into two groups: the TKI group (*n* = 21) and the TKI‐ICI group (*n* = 49). Overall survival (OS) and progression‐free survival (PFS) were analyzed by Kaplan–Meier methods, and multivariate analysis was performed to identify prognostic factors.

**Results:**

In the TKI‐ICI group, median PFS was 5.27 months and median OS was 12.53 months. In the TKI group, median PFS was 3.10 months and median OS was 7.50 months. The objective response rate (ORR) was 19.1% in the TKI group and 16.3% in the TKI‐ICI group. The disease control rate (DCR) was 85.7% in the TKI‐ICI group and 61.9% in the TKI group. In the TKI‐ICI cohort, multivariable Cox analysis revealed the high albumin to neutrophil ratio index (ANRI) was an independent predictor for PFS, while alpha‐fetoprotein > 400 ng/mL was the independent predictor for OS. Safety profiles in both cohorts showed manageable toxicity, with no treatment‐related deaths.

**Conclusions:**

The combination of TKI and ICI presents a promising second‐line treatment option after LEN failure, regardless of the specific second‐line TKI used.

## Graphical abstract

The combination of TKI and ICI for uHCC after LEN failure demonstrated great survival benefits and was well tolerated, irrespective of the second‐line TKI administered.

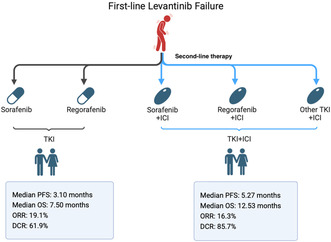

**Abbreviations**
AFP: alpha‐fetoprotein
ALBI: albumin‐bilirubin index
ANRI: albumin to neutrophil ratio index
BCLC: Barcelona Clinic Liver Cancer
CRP: C‐reactive protein
ECOG PS: Eastern Cooperative Oncology Group performance status
HBV: hepatitis B virus
HCV: hepatitis C virus
ICI: immune checkpoint inhibitor
LEN: lenvatinib
LMR: lymphocyte to monocyte ratio
NLR: neutrophil to lymphocyte ratio
PLR: platelet to lymphocyte ratio
PNI: prognostic nutritional index
PVTT: portal vein tumor thrombosis
REG: regorafenib
SIN: sintilimab
SIRI: systemic immune‐inflammation response index
SN: symptom number
SOR: sorafenib
TACE: trans‐arterial chemoembolization
TKI: tyrosine kinase inhibitor

Hepatocellular carcinoma (HCC) is the sixth most prevalent malignant neoplasm worldwide and the fourth leading cause of cancer‐related mortality globally [1]. Approximately 70% of patients are diagnosed at advanced stages, where curative options are limited [2]. Tyrosine kinase inhibitors (TKIs) have become an important therapeutic option for improving the prognosis of patients with uHCC. Sorafenib (SOR) was the first TKI developed for unresectable HCC treatment [3, 4]. Lenvatinib (LEN) has been widely adopted as a first‐line treatment for unresectable HCC due to its demonstrated efficacy in increasing ORR compared to SOR [5, 6]. Despite initial responses, resistance to LEN frequently develops, necessitating effective second‐line therapies.

However, the optimal second‐line treatments for patients with uHCC who have progressed on first‐line LEN treatment have not been clearly defined. Regorafenib (REG) is the first drug approved for the treatment of HCC in patients who have progressed on SOR therapy [7]. It is also accepted as a second‐line treatment following progression on LEN [8]. Additionally, SOR was available as a post‐progression option for patients treated with LEN due to the comparable DCR with the RESORCE study and the CELETIAL study [9]. The advent of immune checkpoint inhibitors (ICIs) has transformed the landscape of second‐line therapy. Huang et al. demonstrated that the oncologic outcomes of REG combined with sintilimab (SIN) as second‐line treatment, the ORR of 36.2%, median OS of 13.4 months, and median PFS of 5.6 months were better than regorafenib monotherapy [10]. Our previous research also indicated that the combined regimen provided greater tumor response and survival benefits than TKI alone [11]. Notably, many existing studies have primarily focused on first‐line SOR failure. Current data on the efficacy and safety of combination therapies as second‐line treatments following LEN failure remain limited [12, 13, 14, 15].

Therefore, we conducted a study to evaluate the efficacy and safety of patients receiving mono‐TKI treatment and those receiving TKI and ICI combination treatment after LEN failure. Furthermore, we investigate the role of inflammation‐related biomarkers as predictive factors, which will help identify the patients who may benefit the most from combination therapy.

## 1 Materials and Methods

### 1.1 Study Design and Patients

This retrospective study encompassed patients who initiated second‐line systemic treatment with TKI or a combination of TKI and ICI (TKI‐ICI) after HCC progressed on LEN between May 2018 and December 2023 at Beijing Ditan hospitals. Eligible participants met the following criteria: (1) age > 18 years; (2) presence of at least one measurable target lesion as defined by modified Response Evaluation Criteria in Solid Tumors (mRECIST) [16]; (3) Eastern Cooperative Oncology Group Performance Status (ECOG PS) of 0–1; (4) Child‐Pugh class A or B (score ≤ 7). Patients were excluded if they had: (1) received any prior systemic therapy other than LEN monotherapy; (2) severe dysfunction of the heart, brain, liver, or kidneys; or (3) coagulation disorders or a high risk of hemorrhage (gastroesophageal varices of grade III, gastric ulcer, and duodenal ulcer). Notably, before the start of the second‐line treatment, all the patients performed upper gastrointestinal endoscopy, and the hemorrhagic risk was assessed. Subsequently, patients were classified based on the type of second‐line treatment administered after HCC progression or discontinuation of LEN due to serious adverse events (AEs).

### 1.2 Treatment Regimens and Assessments

Patients previously treated with LEN (12 mg/day for body weight ≥ 60 kg or 8 mg/day for body weight < 60 kg) received subsequent systemic therapy as determined by their attending specialists upon confirmation of tumor progression or unacceptable toxicity. REG was administered at an initial dose of 80 mg/day in 4‐week cycles (3 weeks on, 1 week off). SOR (400 mg twice daily), donafenib (200 mg twice daily), or apatinib (250 mg three times daily) was administered in 4‐week cycles. Patients also received SIN, camrelizumab, or tislelizumab at 200 mg intravenously on day 1 of a 21‐day cycle following the first REG, SOR, donafenib, or apatinib dose. Treatment continued until disease progression, unacceptable toxicity, or withdrawal by the patient or physician.

Patients received second‐line systemic treatment for 4 weeks per cycle, with treatment response and safety evaluated every 8–12 weeks. Radiological response was assessed according to mRECIST criteria based on liver dynamic computed tomography (CT) or magnetic resonance imaging (MRI) [16]. OS and PFS were calculated from the initiation of second‐line treatment. ORR and disease control rate (DCR) were based on the best radiographic response observed during treatment. ORR was defined as the proportion of patients achieving complete response (CR) and partial response (PR), while DCR included stable disease (SD) in addition to CR and PR. Treatment‐related AEs were assessed according to the Common Terminology Criteria for Adverse Events version 5.0 (CTC‐AE 5.0). The primary endpoint was PFS, and the secondary endpoints included OS, ORR, DCR, and safety.

### 1.3 Variable Collection

Baseline data collected included age, sex, etiology, previous treatment history, ALBI score, and BCLC stage. BCLC stage information encompassed tumor size, number, ECOG PS, portal vein tumor thrombus (PVTT), extrahepatic metastases, and Child‐Pugh class. Besides, the baseline alpha‐fetoprotein (AFP) levels and AFP levels after 8 weeks of treatment were collected.

### 1.4 Inflammation Indices

The analysis included the examination of various inflammation indices, including neutrophil‐to‐lymphocyte ratio (NLR), platelet‐to‐lymphocyte ratio (PLR), prognostic nutritional index (PNI), albumin‐toto‐neutrophil ratio index (ANRI), lymphocyte‐toto‐monocyte ratio (LMR), symptom number (SN), systemic immune‐inflammation response index (SIRI), and C‐reactive protein (CRP). Considering the varying threshold values outlined in the existing literature, we use X‐tile software to determine the cut‐off values (NLR: 5.7; PLR: 197.6; PNI: 403; ANRI: 5.4; LMR: 2.3; SN: 1314.3; SIRI: 4.0; CRP: 46.1 mg/L) and subsequently undertook the process of dichotomizing the index‐related data.

### 1.5 Statistical Analysis

Categorical variables were expressed as numbers (percentages), while continuous variables with skewed distributions were presented as medians [interquartile range]. Baseline characteristics, treatment responses, and AEs between groups were compared using Pearson's *χ* ^2^ test or Fisher's exact test, as appropriate. Survival curves were generated by the Kaplan–Meier method. Factors independently predictive of OS and PFS were evaluated using univariate and multivariate Cox proportional hazards models, incorporating variables with *p* < 0.1 from the univariate analysis into the multivariate analysis. Statistical analyses were conducted using SPSS (version 26.0) and R software (version 4.1.3, http://www.rproject.org), with *p* < 0.05 considered statistically significant.

## 2 Results

### 2.1 Baseline Characteristics

**FIGURE 1:**
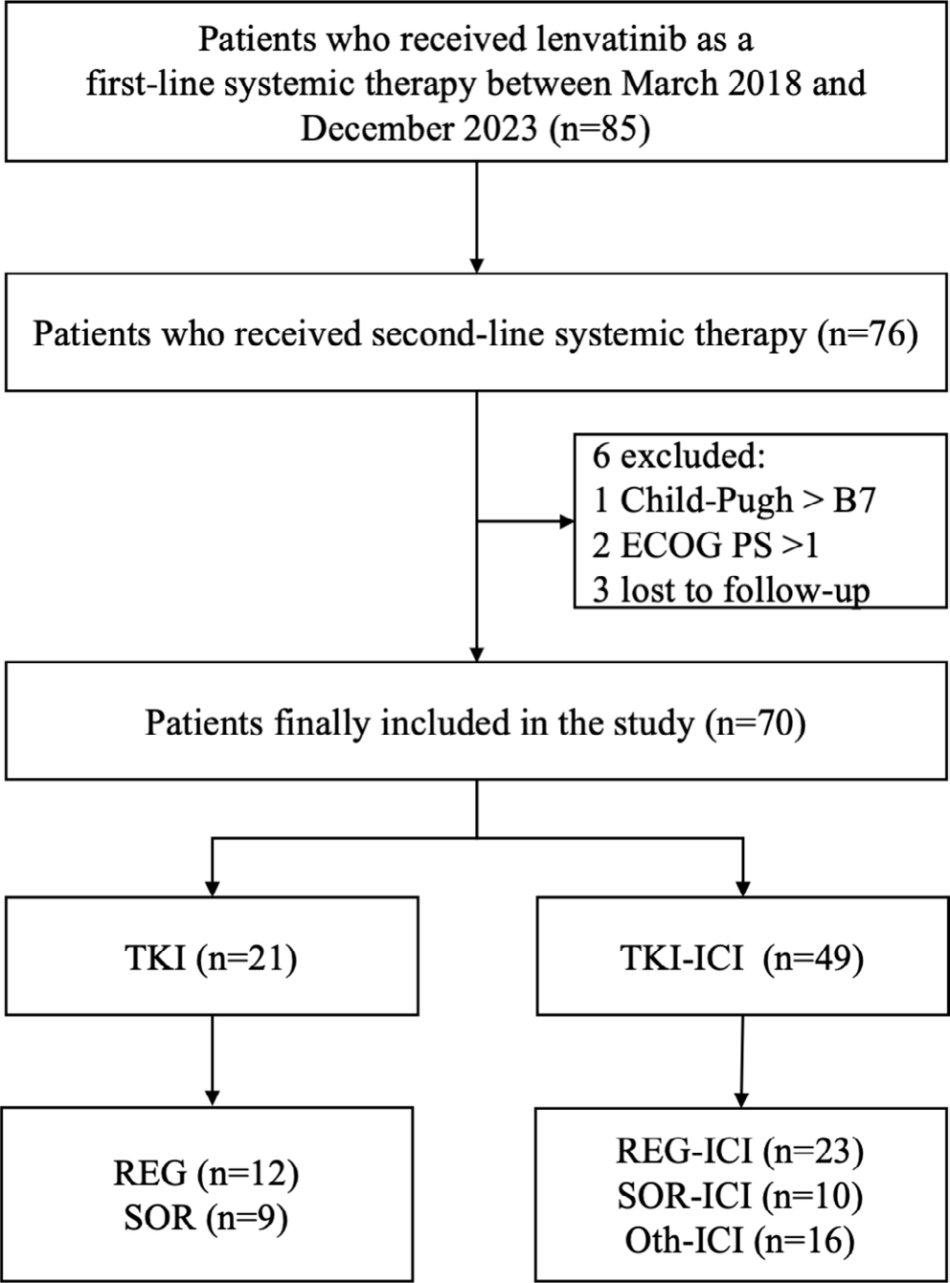
The flow chart of this study.

Between May 2018 and December 2023, a total of 85 HCC patients received LEN as first‐line treatment and subsequently discontinued treatment. Of these, 76 patients who proceeded to second‐line systemic therapy were considered eligible， while 6 patients were excluded for the following reasons: Child‐Pugh score > B7 (*n* = 1), ECOG PS > 1 (*n* = 2), or loss to follow‐up (*n* = 3). Ultimately, a total of 70 patients were enrolled in the study, of whom 21 patients received mono‐TKI treatment and 49 patients received TKI‐ICI combination therapy after LEN failure (Figure 1).

Baseline characteristics are summarized in Table 1. The median age was 56 years (IQR, 51–53), with 59 (84.3%) patients being male. The majority of patients, 63 (90.0%) had HBV‐related HCC, 52 (74.3%) had BCLC stage C tumors, and 47 (67.1%) had Child‐Pugh A liver function. At the start of second‐line therapy, 26 (37.1%) and 34 (48.6%) of patients exhibited the presence of PVTT and extrahepatic metastases, respectively. With regard to prior tumor treatments, 9 (12.9%) patients had undergone hepatectomy, 66 (94.3%) patients had received trans‐arterial chemoembolization (TACE), and 35 (50.0%) patients had undergone ablation. Although the proportion of patients with AFP > 400 ng/mL was higher in the TKI group than in the TKI‐ICI group (52.4% vs. 34.7%, *p* = 0.166), the demographic and disease characteristics of the patients at baseline were balanced between the treatment groups.

**TABLE 1:** Characteristics.

Characteristics	Overall (*n* = 70)	TKI (*n* = 21)	TKI + ICI (*n* = 49)	*p*
Age	56.00 [51.00, 63.00]	54.00 [46.00, 61.00]	56.00 [52.00, 63.00]	0.305
Gender (%)				1
Female	11 (15.7)	3 (14.3)	8 (16.3)	
Male	59 (84.3)	18 (85.7)	41 (83.7)	
Cirrhosis (%)				1
No	7 (10.0)	2 (9.5)	5 (10.2)	
Yes	63 (90.0)	19 (90.5)	44 (89.8)	
Number (%)				0.429
≤ 3	30 (42.9)	11 (52.4)	19 (38.8)	
> 3	40 (57.1)	10 (47.6)	30 (61.2)	
Size (%)				0.602
≤ 5	35 (50.0)	12 (57.1)	23 (46.9)	
> 5	35 (50.0)	9 (42.9)	26 (53.1)	
PVTT (%)				0.483
No	44 (62.9)	15 (71.4)	29 (59.2)	
Yes	26 (37.1)	6 (28.6)	20 (40.8)	
Metastasis (%)				0.876
No	36 (51.4)	10 (47.6)	26 (53.1)	
Yes	34 (48.6)	11 (52.4)	23 (46.9)	
ALBI grade (%)				1
1	18 (25.7)	5 (23.8)	13 (26.5)	
2/3	52 (74.3)	16 (76.2)	36 (73.5)	
AFP (%)				0.166
≤ 400	42 (60.0)	10 (47.6)	32 (65.3)	
> 400	28 (40.0)	11 (52.4)	17 (34.7)	
Etiology (%)				0.778
HBV	63 (90.0)	19 (90.5)	44 (89.8)	
HCV	5 (7.1)	2 (9.5)	3 (6.1)	
Other	2 (2.8)	0 (0.0)	2 (4.0)	
ECOG PS (%)				0.749
0	28 (40)	9 (42.9)	19 (38.8)	
1	42 (60)	12 (57.1)	30 (61.2)	
BCLC stage (%)				0.952
B	18 (25.7)	6 (28.6)	12 (24.5)	
C	52 (74.3)	15 (71.4)	37 (75.5)	
Child‐Pugh class				0.541
A	47 (67.1)	13 (61.9)	34 (69.4)	
B	23 (32.9)	8 (38.1)	15 (30.6)	
Prior treatment				
Surgery (%)	9 (12.9)	2 (9.5)	7 (14.3)	0.876
TACE (%)	66 (94.3)	21 (100.0)	45 (91.8)	0.432
Ablation (%)	35 (50.0)	14 (66.7)	21 (42.9)	0.118
CRP	23.45 [5.58, 36.26]	13.60 [3.80, 36.26]	31.70 [6.30, 36.26]	0.207
ANRI	14.76 [9.17, 24.59]	19.23 [10.49, 30.82]	12.89 [8.89, 22.25]	0.168
LMR	2.60 [1.62, 3.49]	2.29 [1.55, 3.12]	2.62 [1.79, 3.66]	0.302
NLR	2.80 [1.80, 4.77]	3.23 [2.05, 4.91]	2.72 [1.63, 4.52]	0.366
PLR	119.43 [88.84, 181.65]	125.45 [99.51, 182.35]	113.13 [85.16, 179.56]	0.608
PNI	363.51 [315.50, 393.26]	339.00 [307.01, 387.00]	367.00 [317.01, 406.01]	0.431
SN	329.34 [200.10, 669.66]	310.70 [231.93, 686.22]	345.34 [177.93, 619.98]	0.686
SIRI	1.10 [0.60, 2.64]	1.36 [0.69, 2.92]	1.07 [0.58, 2.55]	0.401

*Note:* Values are presented as median (interquartile range) or number (%).

Abbreviations: AFP, alpha‐fetoprotein; ALBI, Albumin‐Bilirubin Index; ANRI, Albumin‐to‐Neutrophil Ratio Index; BCLC, Barcelona Clinic Liver Cancer; CRP, C‐Reactive Protein; ECOG PS, Eastern Cooperative Oncology Group performance status; HBV, hepatitis B virus; HCV, hepatitis C virus; ICI, immune checkpoint inhibitor; LMR, Lymphocyte‐to‐Monocyte Ratio; NLR, Neutrophil‐to‐Lymphocyte Ratio; PLR, Platelet‐to‐Lymphocyte Ratio; PNI, Prognostic Nutritional Index; PVTT, portal vein tumor thrombosis; SIRI, Systemic Immune‐Inflammation Response Index; SN, Symptom Number; TACE, trans‐arterial chemoembolization; TKI, tyrosine kinase inhibitor.

### 2.2 Treatment Profiles of First‐Line LEN and Second‐Line Therapy

Of 70 patients who received first‐line LEN treatment, 55 patients experienced disease progression, and 15 patients discontinued treatment due to adverse events (grade 3/4 bilirubin increase, grade 4 hypertension, grade 3 ALT/AST elevation, and proteinuria). All patients were subsequently transitioned to a second‐line therapeutic regimen. In the second‐line therapy, the TKI group comprised patients receiving REG (*n* = 12) or SOR (*n* = 9). The TKI‐ICI group was divided into three subgroups: REG plus ICI (*n* = 23), SOR plus ICI (*n* = 10), and Oth plus ICI (*n* = 16) (Figure 1).

### 2.3 Survival Analysis

**FIGURE 2:**
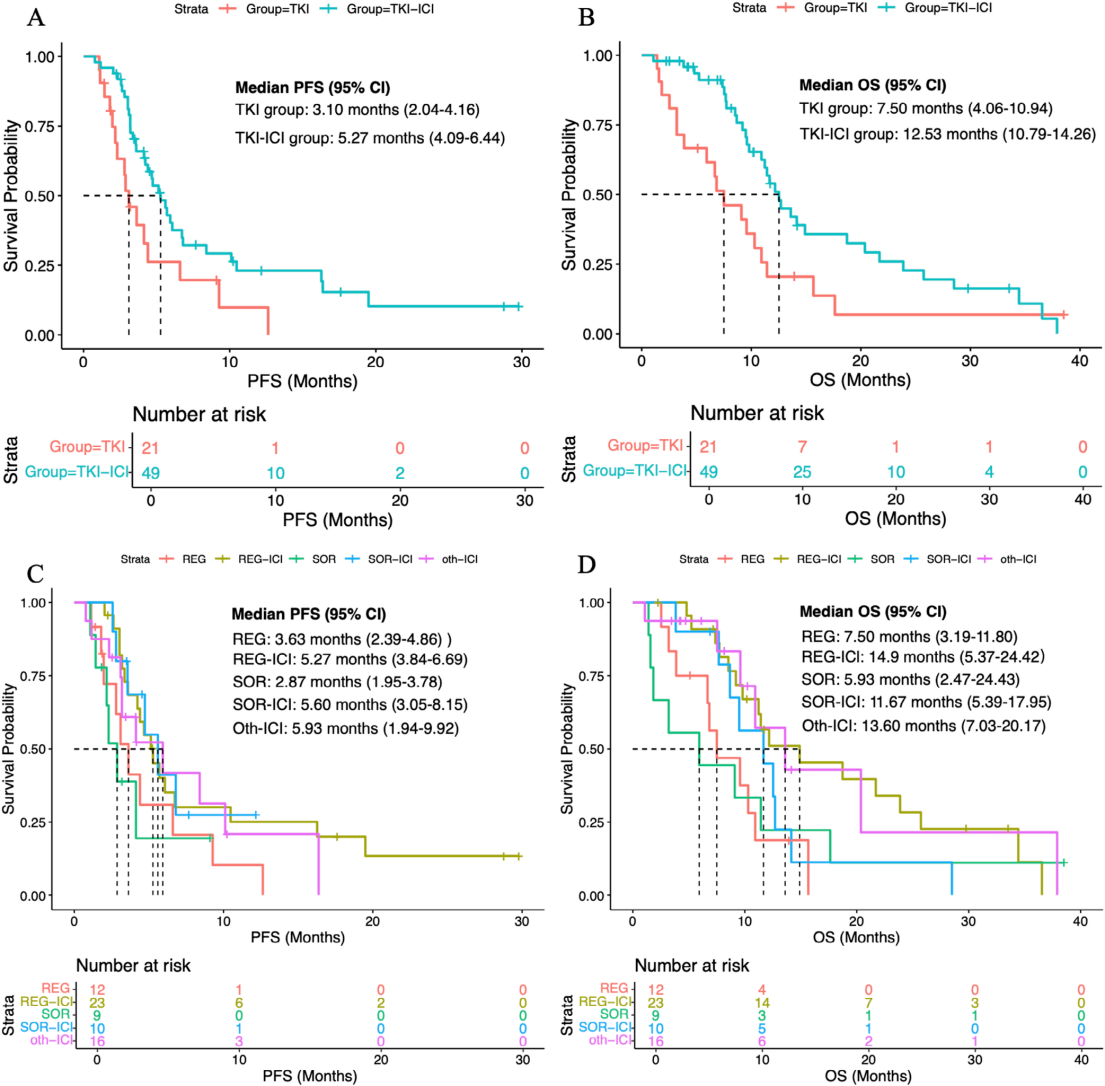
Kaplan–Meier analysis of survival outcomes in patients who received second‐line therapy. (A) Progression‐free survival (PFS) in the TKI group and the TKI‐ICI group. (B) Overall survival (OS) in the TKI group and the TKI‐ICI group. (C) PFS across different second‐line treatment subgroups. (D) OS across different second‐line treatment subgroups.

During the follow‐up duration, 72.9% (*n* = 51) of patients had died, and 72.9% (*n* = 51) of patients had experienced disease progression. The median PFS in the whole population was 4.44 months (95% CI, 3.25–5.55 months), and the median OS was 10.93 months (95% CI, 9.14–12.72 months). The median PFS in the TKI–ICI group was 5.27 months (95% CI, 4.09–6.44 months), and that in the TKI group was 3.10 months (95% CI, 2.04–4.16 months) (Figure 2A). The median OS results were similar to the PFS result, with a median OS of 12.53 months (95% CI, 10.79–14.26 months) for the TKI–ICI group and 7.50 months (95% CI, 4.06–10.94 months) for the TKI group (Figure 2B). The survival curves for the subdivided treatment subgroup are presented in Figure 2C,D, with the respective median PFS and OS detailed in Table 2.

**TABLE 2:** Survival outcomes of second‐line treatment.

Second line	mOS (months)	mPFS (months)
REG (*n* = 12)	7.50 (3.19–11.80)	3.63 (2.39–4.86)
SOR (*n* = 9)	5.93 (2.47–24.43)	2.87 (1.95–3.78)
REG‐ICI (*n* = 23)	14.90 (5.37–24.42)	5.27 (3.84–6.69)
SOR‐ICI (*n* = 10)	11.67 (5.39–17.95)	5.60 (3.05–8.15)
Oth‐ICI (*n* = 16)	13.60 (7.03–20.17)	5.93 (1.94–9.92)
TKI (*n* = 21)	7.50 (4.06–10.94)	3.10 (2.04–4.16)
TKI‐ICI (*n* = 49)	12.53 (10.79–14.26)	5.27 (4.09–6.44)

*Note:* Values are presented as median (interquartile range).

Abbreviations: ICI, immune checkpoint inhibitor; OS, overall survival; PFS, progression‐free survival; REG, regorafenib; SOR, sorafenib; TKI, tyrosine kinase inhibitor.

**FIGURE 3:**
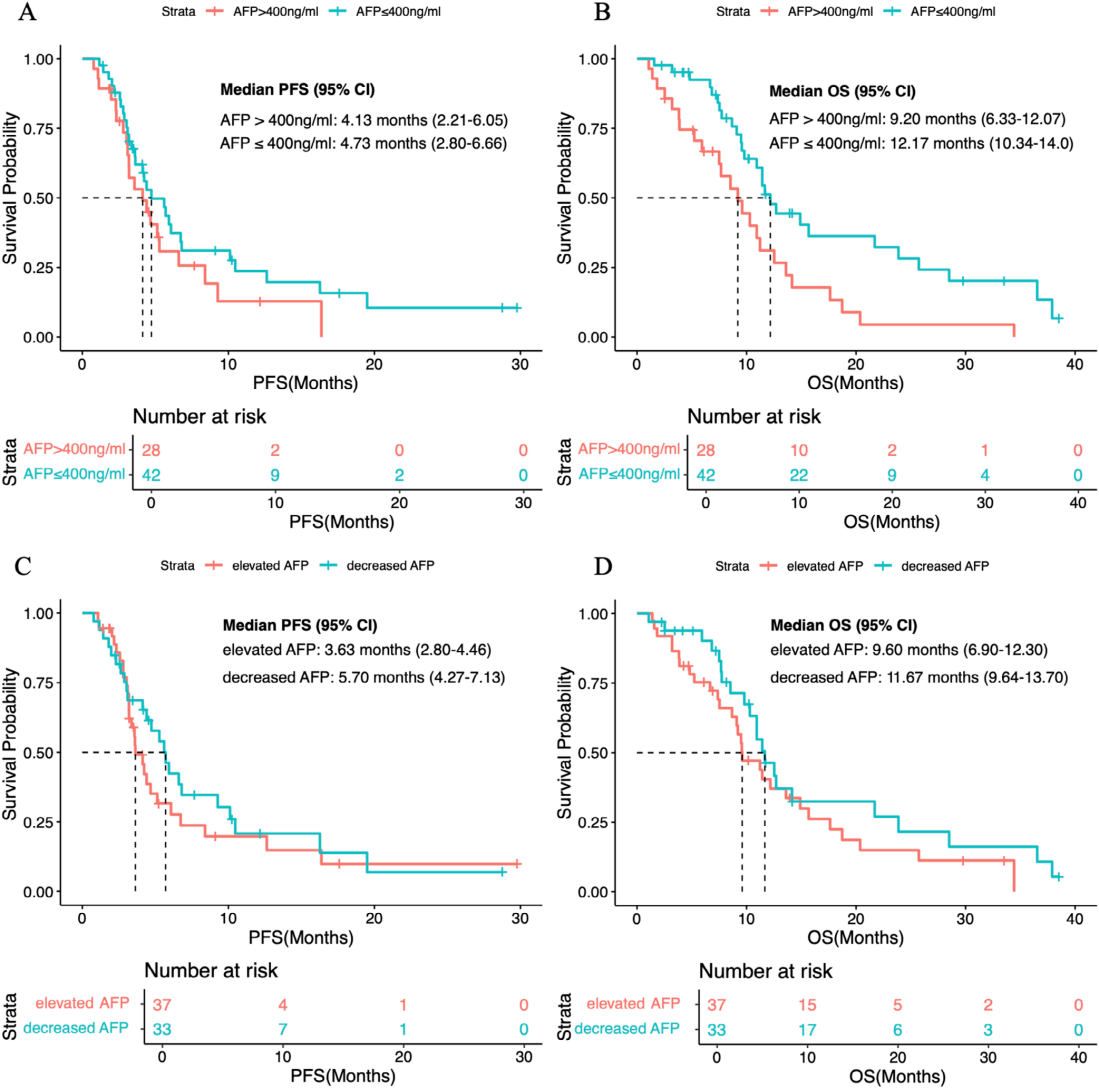
Kaplan–Meier analysis of survival outcomes based on administration of second‐line treatment. (A) PFS between patients treated with SOR and REG. (B) PFS across different combined regimens. (C) OS between patients treated with SOR and REG. (D) OS across different combined regimens.

After 8 weeks of second‐line therapy, 51.0% (*n* = 25) of patients in the TKI–ICI group and 57.1% (*n* = 12) of patients in the TKI group developed elevated AFP levels. When stratified by AFP levels, patients with baseline AFP ≤ 400 ng/mL had a median PFS of 4.73 months (95% CI, 2.80–6.66) and a median OS of 12.17 months (95% CI, 10.34–14.0 months). Patients with AFP > 400 ng/mL had a median PFS of 4.13 months (95% CI, 2.21–6.05) and a median OS of 9.20 months (95% CI, 6.33–12.07 months) (Figure 3A,B). Furthermore, a decrease in AFP levels was not associated with improved survival outcomes (Figure 3C,D).

### 2.4 Therapeutic Responses

**FIGURE 4:**
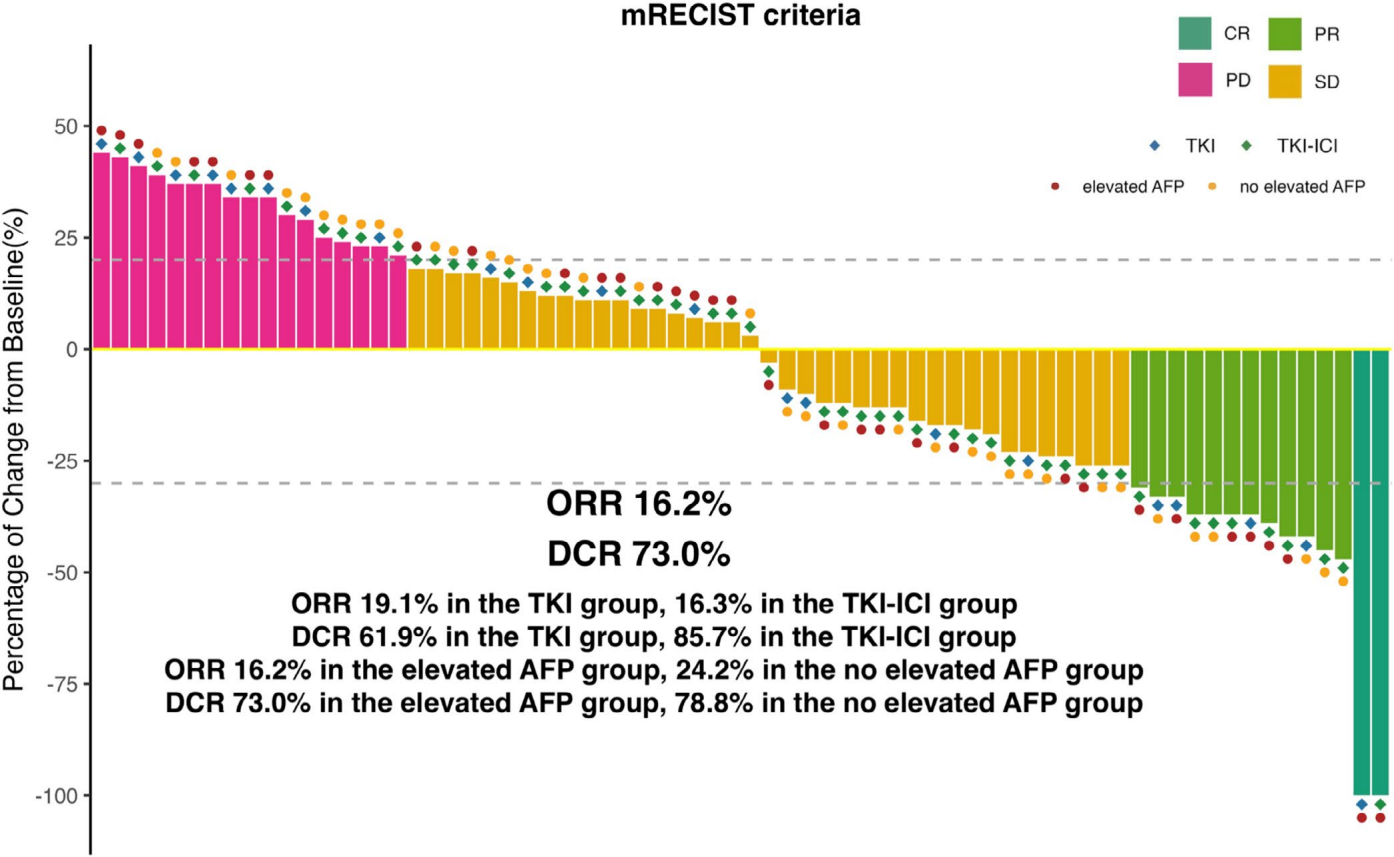
Waterfall plot of maximum tumor response to different second‐line treatment modalities and AFP levels after 8 weeks of treatment.

Therapeutic responses to second‐line treatments are summarized in Table 3, as assessed by mRECIST criteria. Four (19.1%) patients in the TKI group had a confirmed ORR, including 1 (4.8%) patient with CR and 3 (14.3%) with PR. In the TKI‐ICI group, the ORR was 16.3%, including 1 (2%) patient who achieved CR and 7 (14.3%) patients who achieved PR. The DCR was 85.7% in the TKI‐ICI group and 61.9% in the TKI group. After 8 weeks of second‐line treatment, patients with decreased AFP levels exhibited an ORR of 24.2% and a DCR of 78.8%, while those with elevated AFP levels showed an ORR of 16.2% and a DCR of 73.0% (Figure 4). This result demonstrated that the pattern of serologic response was not associated with the mRECIST response.

**TABLE 3:** Tumor response.

	TKI (*n* = 21)	TKI + ICI (*n* = 49)	*p*
Best overall response			
Complete response	1 (4.8)	1 (2)	0.513
Partial response	3 (14.3)	7 (14.3)	0.942
Stable disease	9 (42.9)	34 (69.4)	0.037
Progressive disease	8 (38.1)	7 (14.3)	0.026
Objective response rate	4 (19.1)	8 (16.3)	0.743
Disease control rate	13 (61.9)	42 (85.7)	0.026

*Note:* Data are presented as number (%) or % as appropriate.

Abbreviations: ICI, immune checkpoint inhibitor; mRECIST, modified response evaluation criteria in solid tumors; TKI, tyrosine kinase inhibitor.

### 2.5 Predictors for Survival Outcomes

Predictors for survival outcomes are depicted in Table 4.

**TABLE 4:** Cox regression for PFS and OS in the whole cohort.

Characteristics	PFS				OS			
	Univariate analysis		Multivariate analysis		Univariate analysis		Multivariate analysis	
	HR (95% CI)	*p*	HR (95% CI)	*p*	HR (95% CI)	*p*	HR (95% CI)	*p*
Age (> 60 vs. ≤ 60)	0.66 (0.37–1.18)	0.160			0.64 (0.36–1.17)	0.148		
Cause (HBV vs. others)	0.55 (0.23–1.33)	0.185			0.72 (0.32–1.62)	0.419		
Surgery (yes or no)	0.90 (0.40–2.03)	0.801			0.70 (0.31–1.57)	0.388		
TACE (yes or no)	0.47 (0.14–1.55)	0.215			0.83 (0.2–3.48)	0.805		
Ablation (yes or no)	0.68 (0.38–1.23)	0.208			0.67 (0.38–1.19)	0.173		
BCLC stage (C vs. B)	1.44 (0.72–2.90)	0.305			0.87 (0.45–1.71)	0.682		
Cirrhosis (yes or no)	1.06 (0.42–2.69)	0.897			1.11 (0.43–2.85)	0.837		
Number (> 3 vs. ≤ 3)	1.36 (0.78–2.38)	0.282			1.51 (0.85–2.67)	0.162		
Size (> 5 cm vs. ≤ 5 cm)	1.08 (0.62–1.88)	0.790			1.20 (0.69–2.08)	0.516		
PVTT (yes or no)	1.00 (0.56–1.8)	1.000			0.96 (0.54–1.70)	0.879		
Metastasis (yes or no)	0.92 (0.53–1.60)	0.769			1.48 (0.81–2.69)	0.198		
Child‐Pugh class (B vs. A)	1.18 (0.65–2.16)	0.590			1.23 (0.68–2.21)	0.491		
ALBI Grade (2/3 vs. 1)	1.01 (0.55–1.85)	0.978			1.45 (0.72–2.91)	0.297		
AFP (> 400 vs. ≤ 400)	1.44 (0.82–2.54)	0.207			2.22 (1.25–3.93)	0.006	2.04 (1.10–3.79)	0.023
Gender (male vs. female)	1.06 (0.50–2.28)	0.873			0.89 (0.40–1.99)	0.773		
Second‐line (TKI‐ICI vs. TKI)	0.48 (0.26–0.88)	0.017	0.49 (0.26–0.95)	0.033	0.52 (0.29–0.93)	0.025	0.64 (0.33–1.22)	0.173
ECOG PS (1 vs. 0)	1.39 (0.79–2.47)	0.256			1.61 (0.91–2.83)	0.100		
CRP (high vs. low)	2.10 (0.97–4.53)	0.06	1.04 (0.37–2.93)	0.943	1.57 (0.69–3.55)	0.280		
ANRI (high vs. low)	0.28 (0.12–0.65)	0.003	0.23 (0.08–0.62)	0.004	1.59 (0.56–4.49)	0.390		
LMR (high vs. low)	0.45 (0.25–0.82)	0.009	0.80 (0.37–1.73)	0.572	0.47 (0.27–0.84)	0.010	0.69 (0.35–1.37)	0.284
NLR (high vs. low)	2.51 (1.14–5.51)	0.022	1.91 (0.66–5.48)	0.231	2.55 (1.21–5.39)	0.014	0.94 (0.35–2.51)	0.941
PLR (high vs. low)	1.79 (0.94–3.44)	0.079	1.37 (0.53–3.54)	0.51	1.40 (0.70–2.79)	0.346		
PNI (high vs. low)	0.46 (0.23–0.94)	0.033	0.53 (0.24–1.18)	0.119	0.49 (0.24–1.01)	0.054	0.57 (0.26–1.26)	0.164
SN (high vs. low)	2.23 (1.02–4.88)	0.044	0.47 (0.08–0.62)	0.402	2.18 (0.95–4.97)	0.065	1.92 (0.70–5.23)	0.205
SIRI (high vs. low)	2.45 (1.15–5.22)	0.02	1.60 (0.34–7.46)	0.55	1.63 (0.68–3.91)	0.277		

Abbreviations: AFP, alpha‐fetoprotein; ALBI, Albumin‐Bilirubin Index; ANRI, Albumin to Neutrophil Ratio Index; BCLC, Barcelona Clinic Liver Cancer; CRP, C‐Reactive Protein; ECOG PS, Eastern Cooperative Oncology Group performance status; HBV, hepatitis B virus; HR, hazard rate; ICI, immune checkpoint inhibitor; LMR, Lymphocyte to Monocyte Ratio; NLR, Neutrophil to Lymphocyte Ratio; OS, overall survival; PFS, progression‐free survival; PLR, Platelet to Lymphocyte Ratio; PNI, Prognostic Nutritional Index; PVTT, portal vein tumor thrombosis; SIRI, Systemic Immune‐Inflammation Response Index; SN, Symptom Number; TACE, trans‐arterial chemoembolization; TKI, tyrosine kinase inhibitor.

Univariable and multivariate Cox regression analyses identified AFP > 400 ng/mL (HR 2.04; 95% CI 1.10–3.79; *p* = 0.03) as the sole independent predictor for OS. Significant predictors for PFS in univariable analysis included second‐line treatment modality, CRP, ANRI, LMR, NLR, PLR, PNI, SN, and SIRI. Multivariable analysis revealed that TKI‐ICI treatment (HR 0.49; 95% CI 0.26–0.95; *p* = 0.033) and high ANRI (HR 0.23; 95% CI 0.08–0.26; *p* = 0.004) were protective predictors of HCC progression. Furthermore, in the TKI‐ICI population, AFP (HR 2.68; 95% CI 1.26–5.70; *p* = 0.01) remained an independent predictor for OS, and high ANRI (HR 0.27; 95% CI 0.08–0.87; *p* = 0.029) was also associated with improved PFS (Table 5).

**TABLE 5:** Cox regression for PFS and OS in the TKI‐ICI cohort.

Characteristics	PFS				OS			
	Univariate analysis		Multivariate analysis		Univariate analysis		Multivariate analysis	
	HR (95% CI)	*p*	HR (95% CI)	*p*	HR (95% CI)	*p*	HR (95% CI)	*p*
Age (> 60 vs. ≤ 60)	0.60 (0.30–1.20)	0.146			0.83 (0.40–1.70)	0.607		
Cause (HBV vs. others)	0.39 (0.11–1.36)	0.141			0.88 (0.34–2.05)	0.691		
Surgery (yes or no)	1.02 (0.39–2.69)	0.971			0.65 (0.24–1.75)	0.393		
TACE (yes or no)	3.26 (0.95–11.20)	0.061	0.49 (0.13–1.83)	0.291	0.53 (0.12–2.30)	0.399		
Ablation (yes or no)	0.37 (0.17–0.79)	0.010	0.51 (0.19–1.36)	0.178	0.51 (0.24–1.07)	0.075	0.68 (0.23–2.06)	0.50
BCLC stage (C vs. B)	1.52 (0.62–3.74)	0.362			1.26 (0.48–3.34)	0.642		
Cirrhosis (yes or no)	1.10 (0.39–3.15)	0.854			2.34 (0.54–10.08)	0.897		
Number (> 3 vs. ≤ 3)	0.64 (0.33–1.27)	0.204			1.77 (0.84–3.77)	0.136		
Size (> 5 cm vs. ≤ 5 cm)	1.47 (0.74–2.95)	0.275			0.94 (0.46–1.88)	0.855		
PVTT (yes or no)	0.97 (0.49–1.93)	0.934			1.34 (0.65–2.76)	0.427		
Metastasis (yes or no)	1.08 (0.55–2.11)	0.824			1.55 (0.70–3.42)	0.280		
Child‐Pugh class (B vs. A)	0.70 (0.33–1.50)	0.358			1.88 (0.89–3.99)	0.099	1.51 (0.65–3.49)	0.339
ALBI grade (2/3 vs. 1)	1.04 (0.49–2.18)	0.925			1.18 (0.51–2.75)	0.701		
AFP (> 400 vs. ≤ 400)	1.28 (0.63–2.60)	0.491			2.11 (1.03–4.32)	0.042	2.68 (1.26–5.70)	0.010
Gender (male vs. female)	1.01 (0.42–2.45)	0.980			1.06 (0.39–3.05)	0.915		
ECOG PS (1 vs. 0)	1.42 (0.71–2.83)	0.324			1.47 (0.73–2.98)	0.285		
AFP	1.72 (0.87–3.40)	0.116			1.40 (0. 68–2.85)	0.363		
CRP (high vs. low)	2.02 (0.77–5.30)	0.152			1.28 (0.44–3.78)	0.650		
ANRI (high vs. low)	0.27 (0.10–0.74)	0.011	0.27 (0.08–0.87)	0.029	0.69 (0.20–2.34)	0.552		
LMR (high vs. low)	0.45 (0.22–0.93)	0.030	1.34 (0.48–3.75)	0.578	0.54 (0.26–1.11)	0.095	0.77 (0.25–2.36)	0.652
NLR (high vs. low)	2.01 (0.69–5.88)	0.200			3.83 (1.21–12.10)	0.022	3.23 (0.83–12.56)	0.090
PLR (high vs. low)	1.71 (0.78–3.73)	0.180			1.65 (0.68–4.00)	0.270		
PNI (high vs. low)	0.40 (0.18–0.90)	0.028			0.54 (0.24–1.21)	0.136		
SN (high vs. low)	2.12 (0.79–5.63)	0.134			2.06 (0.69–6.11)	0.193		
SIRI (high vs. low)	2.59 (1.03–6.54)	0.043	1.79 (0.60–5.37)	0.296	1.31 (0.39–4.45)	0.664		

Abbreviations: AFP, alpha‐fetoprotein; ALBI, Albumin‐Bilirubin Index; ANRI, Albumin to Neutrophil Ratio Index; BCLC, Barcelona Clinic Liver Cancer; CRP, C‐Reactive Protein; ECOG PS, Eastern Cooperative Oncology Group performance status; HBV, hepatitis B virus; HR, hazard rate; ICI, immune checkpoint inhibitor; LMR, Lymphocyte to Monocyte Ratio; NLR, Neutrophil to Lymphocyte Ratio; OS, overall survival; PFS, progression‐free survival; PLR, Platelet to Lymphocyte Ratio; PNI, Prognostic Nutritional Index; PVTT, portal vein tumor thrombosis; SIRI, Systemic Immune‐Inflammation Response Index; SN, Symptom Number; TACE, trans‐arterial chemoembolization; TKI, tyrosine kinase inhibitor.

### 2.6 Safety Analysis

Treatment‐related adverse events of any grade occurred in 79.6% of patients in the TKI‐ICI arm and 85.7% in the TKI arm, the most common of which were hypertension, diarrhea, nausea, and vomiting. A total of 9 patients (18.4%) in the TKI‐ICI group and 5 (23.8%) in the TKI group experienced at least one grade ≥ 3 AE. It is noteworthy that immune‐related AEs in the TKI‐ICI group included two cases of immune hepatitis, one case of immune myocarditis, and one case of interstitial pneumonia. Additionally, 7 patients (14.3%) in the TKI‐ICI group and 3 (14.3%) in the TKI group experienced AEs leading to dose reduction. The proportion of patients who discontinued treatment due to AEs was 8.2% in the TKI‐ICI group and 4.8% in the TKI group (Table 6).

**TABLE 6:** Adverse events.

Adverse event	Any grade			Grade 3/4		
	TKI group (*n* = 21)	TKI‐ICI group (*n* = 49)	*p*	TKI group (*n* = 21)	TKI‐ICI group (*n* = 49)	*p*
**Total AEs**	**18 (85.7)**	**39 (79.6)**	**0.741**	**5 (23.8)**	**9 (18.4)**	**0.745**
Hypertension	14 (66.7)	32 (65.3)		2 (9.5)	6 (12.2)	
Fatigue	6 (28.6)	13 (26.5)		1 (4.8)	1 (2.0)	
Nausea/vomiting	4 (29.7)	15 (30.6)		0 (0.0)	2 (4.1)	
Diarrhea	7 (33.3)	11 (22.4)		1 (4.8)	1 (2.0)	
Rash	3 (14.3)	9 (18.4)		0 (0.0)	1 (2.0)	
Decreased appetite	3 (14.3)	7 (14.3)		1 (4.8)	1 (2.0)	
Proteinuria	2 (9.5)	3 (6.1)		0 (0.0)	0 (0.0)	
Leukopenia	3 (14.3)	5 (10.2)		1 (4.8)	0 (0.0)	
Hypothyroidism	2 (9.5)	3 (6.1)		0 (0.0)	1 (2.0)	
Ascites	3 (14.3)	2 (4.1)		0 (0.0)	0 (0.0)	
Hyperbilirubinemia	1 (4.8)	1 (2.0)		0 (0.0)	1 (2.0)	
Thin	0 (0.0)	1 (2.0)		0 (0.0)	0 (0.0)	
Thrombocytopenia	1 (4.8)	2 (4.1)		0 (0.0)	1 (2.0)	
Hepatic encephalopathy	1 (4.8)	2 (4.1)		1 (4.8)	2 (4.1)	
Gastrointestinal hemorrhage	0 (0.0)	1 (2.0)		0 (0.0)	0 (0.0)	
**Immune‐related AEs**	**0 (0.0)**	**2 (4.1)**	**1**	**0 (0.0)**	**2 (4.1)**	**1**
Myocarditis	0 (0.0)	1 (2.0)		0 (0.0)	1 (2.0)	
Interstitial pneumonia	0 (0.0)	1 (2.0)		0 (0.0)	1 (2.0)	
**Dose reduction**	**3 (14.3)**	**7 (14.3)**	**1**	**3 (14.3)**	**7 (14.3)**	**1**
**Discontinuation**	**1 (4.8)**	**4 (8.2)**	**1**	**1 (4.8)**	**4 (8.2)**	**1**

*Note:* Data are presented as number (%) or % as appropriate.

Abbreviations: AE, adverse event; ICI, immune checkpoint inhibitor; TKI, tyrosine kinase inhibitor.

### 2.7 Subsequent Therapy

Among the TKI group, 5 (25%) patients received subsequent systemic treatment, including apatinib (*n* = 1), REG plus SIN/camrelizumab (*n* = 3), and LEN plus SIN (*n* = 1). 15 (75%) patients in the TKI‐ICI group performed a 3rd line systemic treatment, with options including apatinib (*n* = 2), apatinib plus SIN/cadonilimab/tislelizumab (*n* = 5), LEN (*n* = 1), LEN plus SIN/Tislelizumab (*n* = 2), REG plus camrelizumab (*n* = 3), and atezolizumab plus bevacizumab (*n* = 2).

## 3 Discussion

In China, LEN is widely used as the first‐line treatment of patients with HCC due to its relatively low risk of gastrointestinal bleeding and cost‐effectiveness [5, 17]. However, its clinical efficacy is often limited by drug resistance, poor tolerance, and serious AEs, making effective second‐line treatments crucial for improving the survival of patients with advanced HCC [7, 18, 19]. A significant limitation of LEN therapy is the scarcity of effective post‐treatment options after its failure [20, 21]. Current guidelines from the China National Liver Cancer (CNLC) recommend REG, apatinib, camrelizumab, and tislelizumab as second‐line systemic therapies [22]. However, most phase III trials were designed with SOR as the standard first‐line treatment, limiting their relevance to patients who have progressed on LEN.

The study is the first to evaluate the effectiveness of SOR‐ or REG‐based monotherapy or combination therapy in patients with uHCC who had progressed on LEN. The findings supported the potential of combining TKI and ICI as a second‐line option after LEN failure. In our cohort, TKI‐ICI therapy significantly improved tumor response, achieving disease control in 85.7% of patients. Furthermore, the survival outcomes were favorable, with a median PFS of 5.27 months in the TKI‐ICI group and 3.10 months in the TKI group. A similar trend was observed in OS, with a median OS of 12.53 months in the TKI‐ICI group and 7.50 months in the TKI group. Multivariate analyses further identified that TKI combined with ICI was a significant predictor of improved PFS, while AFP > 400 ng/mL was associated with inferior OS.

Regorafenib has been commonly used for advanced HCC patients following sorafenib [7]. The median PFS in our TKI group aligns with the outcomes of the RESORCE trial. Recent clinical data suggested that regorafenib remains effective in advanced HCC patients, whereas sorafenib may not be a suitable post‐treatment option after LEN [8, 23]. Koroki et al. found that PFS, ORR, and DCR in patients treated with sorafenib after LEN were 1.8 months, 1.8%, and 20.8%, respectively—markedly worse than those reported in phase III studies of other second‐line systemic therapy [18, 23, 24]. However, in our study, the second‐line mono‐TKI group showed similar OS and PFS outcomes for SOR and REG, consistent with the findings of Tomonari et al., who demonstrated that SOR was non‐inferior to REG in terms of PFS, ORR, and DCR [9]. Both SOR and REG are known to inhibit platelet‐derived growth factor receptor (PDGFR) and KIT more effectively than LEN, which may account for the comparable clinical outcomes observed following LEN therapy [25].

The synergistic effect of combining TKIs and ICIs, attributed to VEGF signaling inhibition and tumor immune microenvironment transformation, has been supported by clinical trials demonstrating superior efficacy for advanced HCC as a first‐line treatment [12, 26, 27, 28]. The RESCUE trial was the first to report excellent outcomes of TKI plus ICI as a second‐line therapy for advanced HCC [27]. Additionally, Huang et al. demonstrated that REG plus SIN provided greater survival benefits than REG monotherapy [10]. Although only 20 patients (36.4%) had received LEN as first‐line treatment, subgroup analysis revealed that those treated with first‐line LEN had a lower risk of death with second‐line REG plus SIN compared to second‐line REG alone. Our findings emphasize the efficacy of combined therapy, regardless of the second‐line TKI used. Guan et al. [29] further reported that LEN plus ICI achieved a median PFS of 8.7 months and a higher DCR of 82.7%, surpassing the outcomes of REG monotherapy. These collective findings support the potential of TKI–ICI therapy after LEN failure.

The presence of tumor‐associated inflammation represents a promotion of tumorigenesis and progression [30]. Circulating inflammatory markers could reflect the underlying systemic inflammation and provide prognostic information for HCC [31]. In our TKI‐ICI cohort, high ANRI was identified as a protective predictor, associated with greater PFS. Previous studies have shown that neutrophils promote tumor invasion, metastasis, and angiogenesis through the release of tumor suppression factors, hepatocyte growth factor, neutrophil elastase, and matrix metalloproteins [32]. Conversely, AFP > 400 ng/mL was an adverse predictor of inferior OS. AFP is indicative of a biologically distinct subtype of HCC, often associated with poor prognosis and more stem‐cell‐like features (such as Epithelial Cell Adhesion Molecule [Ep‐CAM] expression), increased vascular endothelial growth factor (VEGF) pathway activity, and increased activity of VEGFR2‐targeted antibodies in preclinical models [33, 34]. Interestingly, in our study, decreased AFP levels following second‐line treatment were not correlated with patient prognosis or tumor response. To date, the approval of ramucirumab in patients receiving second‐line treatment with an AFP greater than 400 ng/mL represents the only biomarker‐indicated approval in HCC [18]. Regrettably, it is not widely available for use in our research center.

The safety profile observed in our study was consistent with the previous study, with no new safety concerns emerging. The most common AEs in the TKI group included hypertension, diarrhea, nausea, and vomiting. The TKI‐ICI combination did not significantly increase the incidence of AEs. 14.3% of patients in both groups required dose reduction due to TKI‐related AEs. Most AEs were generally manageable, and the rate of AEs leading to treatment discontinuation was similar between the TKI‐ICI and TKI groups.

Despite these promising results, the study is not without limitations. The retrospective design and single‐center data collection may introduce selection bias (e.g., 90.0% of patients with HBV‐related hepatopathy) and limit the generalizability of the findings. Additionally, the relatively small sample size and insufficient follow‐up duration necessitate cautious interpretation of the results. Thirdly, the efficacy of ICI monotherapy was not analyzed. Future prospective studies with larger patient populations and randomized controlled designs are warranted to validate these findings.

## 4 Conclusions

The combination of TKI and ICI presents a promising second‐line treatment option after LEN failure, regardless of the specific second‐line TKI used. Our study demonstrated improved tumor control and survival outcomes with this approach, highlighting its potential role in the management of advanced HCC. However, future studies with larger sample sizes, longer follow‐up periods, and a multicenter design are necessary to confirm these findings and to optimize second‐line treatment strategies. Additionally, further exploration of predictive biomarkers such as AFP and inflammatory markers may help guide personalized treatment approaches, improving outcomes for patients with advanced HCC.

## Author Contributions

**Xue Yin:** data curation (equal), methodology (equal), software (equal), visualization (equal), writing – original draft (equal), writing – review and editing (equal). **Na Deng:** formal analysis (equal), visualization (equal), writing – original draft (equal), writing – review and editing (equal). **Jinglong Chen:** conceptualization (equal), writing – original draft (equal), writing – review and editing (equal). **Xiaoyan Ding:** conceptualization (equal), visualization (equal), writing – original draft (equal), writing – review and editing (equal).

## Ethics Statement

The study was approved by the Ethics Committee of Beijing Ditan Hospital, Capital Medical University (Approval number: DTEC‐KY2022‐014‐01) and complied with the Declaration of Helsinki and clinical practice guidelines. Informed written consent was obtained from all study participants prior to enrollment.

## Conflicts of Interest

The authors declare no conflicts of interest.

## Data Availability

The dataset utilized in this study is available from the corresponding author upon reasonable request.
